# Exogenous Lipoxin A4 attenuates IL4-induced Mucin Expression in Human Airway Epithelial Cells

**DOI:** 10.7150/ijms.79525

**Published:** 2023-02-05

**Authors:** Eun-Hye Seo, Seung Hyun Lee, Bo Yoon Choi, Chung-Sik Oh, Jin Kook Kim

**Affiliations:** 1 BKPlus21, Department of Microbiology, School of medicine, Konkuk University, Seoul, Korea; 2 Departments of Otorhinolaryngology-Head & Neck Surgery, School of medicine, Konkuk University, Seoul, Korea; 3 Department of Anesthesiology and Pain Medicine, Konkuk University School of Medicine, Konkuk University Medical Center, Seoul, Korea

**Keywords:** Lipoxin A4, IL-4, MUC5AC, MUC5B, MAPK

## Abstract

**Introduction:** The proinflammatory cytokine interleukin-4 (IL-4) induces mucus hypersecretion by human airway epithelial cells and the MAP kinase signalling pathway may be important in terms of IL-4-induced MUC5AC gene expression. Lipoxin A_4_ (LXA_4_) is an arachidonic acid-derived mediator that promotes inflammation by binding to the anti-inflammatory receptors (ALXs) or the formyl-peptide receptor like-1 (FPRL1) protein expressed by airway epithelial cells. Here, we explore the effects of LXA4 on IL-4-induced mucin gene expression in, and secretion from, human airway epithelial cells.

**Methods:** We co-treated cells with IL-4 (20 ng/mL) and LXA_4_ (1 nM) and measured the expression levels of mRNAs encoding MUC5AC and 5B via real-time polymerase chain reaction; protein expression levels were determined by Western blotting and immunocytofluorescence. The ability of IL-4 and LXA_4_ to suppress protein expression was determined by Western blotting.

**Results:** IL-4 increased MUC5AC and 5B gene and protein expression. LXA_4_ suppressed IL-4-induced MUC5AC and 5B gene and protein expression by interacting with the IL4 receptor and mitogen-activated protein kinase (MAPK) pathway, including both phospho-p38 MAPK and phospho-extracellular signal-regulated kinase (phospho-ERK). IL-4 and LXA_4_ increased and decreased, respectively, the number of cells that stained with anti-MUC5AC and 5B antibodies.

**Conclusions:** LXA_4_ may regulate mucus hypersecretion induced by IL4 in human airway epithelial cells.

## Introduction

Lipoxin (LX) A4 (LXA_4_) is an arachidonic acid-derived metabolite produced by the 12/15-lipoxygenase (12/15-LO) enzymes that are generally associated with anti-inflammatory effects 1-3. LXA_4_ is usually synthesised during interactions between leukocytes and resident cells that develop in response to inflammation; it prevents an excessive inflammatory response, thus limiting damage to the host. Several studies have shown that the various LXs exhibit potent anti-inflammatory actions in patients with respiratory diseases such as asthma and allergic conditions 4. LXA_4_ exerts its biological actions via the specific high-affinity G-protein-coupled receptor formyl-peptide receptor like-1 (FPRL-1) protein and anti-inflammatory receptors (ALXs), which are expressed by both human airway epithelial cells and leukocytes. The synthesis of ALXs is induced by specific inflammatory mediators 5.

Mucus hypersecretion by the airway epithelium is a major feature of several respiratory diseases 6,7. MUC5AC and MUC5B are generally considered to be the major airway mucins 8. Of the various mucins, the levels of mRNAs encoding MUC5 AC and MUC5 B, and the levels of the proteins, were significantly increased in patients with chronic rhinosinusitis (CRS) and allergic rhinitis compared to subjects with normal sinus mucosa 9,10. Th2 cytokines, including interleukin (IL)-4, IL-9, and IL-13, play major roles in allergic inflammation. Th2-mediated inflammation is involved in the allergic responses of multiple sites; Th2 cells interact with epithelial cells to change the airway microenvironment 11. The allergic pulmonary inflammation and airway hyper-reactivity associated with asthma are attributable to signalling by the Th2 cytokines IL4 and IL13, and require the IL4 receptor-α-chain 12. These cytokines increased MUC5AC expression in human cultured epithelial cells 13,14. However, any effect of LXA_4_ on IL4-stimulated mucin expression by airway epithelial cells remains unclear. We hypothesized that LXA_4_ might also attenuates the expression of the major airway mucins (MUC5AC and MUC5B), thus regulating cell signalling. We first explored whether LXA_4_ suppressed the IL-4-induced expression of MUC5AC and MUC5B, which are the major secreted mucins. We also explored how LXA_4_ inhibited the signals controlling MUC5AC and MUC5B gene and protein expression in human airway epithelium.

## Materials and Methods

### Materials

LXA_4_ and IL-4 were obtained from Millipore (Burlington, MA, USA). Primary human nasal epithelial (NHNE) cells and airway epithelial cell growth medium were purchased from PromoCell (Heidelberg, Germany). RPMI 1640 medium and TRIzol were purchased from Invitrogen (Carlsbad, CA, USA). Real-time PCR kits were obtained from Roche Applied Science (Mannheim, Germany). A 21-nucleotide sequence siRNA corresponding to the human FPR1 sequence (5′-AGAAAUUGGUAUUGCAGUGUU-3′) were purchased from Bioneer (Daejeon, South Korea). Primary and secondary antibodies targeting MUC5AC and MUC5B (used in immunoassays) were obtained from Santa Cruz Biotechnology (Santa Cruz, CA, USA). Antibodies against p38, extracellular signal-regulated kinase (ERK), JNK, and phospho-p38 were from Cell Signaling Technology (Danvers, MA, USA). Foetal bovine serum (FBS) was purchased from Hyclone Laboratories (Logan, UT, USA). This study was approved by the Institutional Review Board of Konkuk University Medical Center (Seoul, Republic of Korea; approval no. KUH-1110016).

### Cell cultures

The human bronchial epithelial cell line (BEAS 2B) was purchased from the American Type Culture Collection (Manassas, VA, USA; catalogue no. CRL-1848). The cells were maintained in Dulbecco's modified Eagle's medium (DMEM) supplemented with 10% (v/v) heat-inactivated FBS and 1% (w/v) penicillin in 10-cm-diameter dishes. The medium was replaced every 3 days. NHNE cells were cultured as previously described 9 and, at passage 2, were seeded (1 × 10^5^ cells/well) in 0.5-mL amounts of culture medium into 12 Transwell clear culture inserts (0.45-mm pore diameter; Costar Co., Cambridge, MA, USA) and cultured in a 1:1 (v/v) mixture of basal epithelial growth medium and DMEM containing the supplements described above at 37ºC under 5% (v/v) CO_2_. The cells were grown submerged until they reached confluence. Full differentiation of bronchial epithelial cells was usually evident after 2 weeks.

### siRNA inhibition

BEAS2B and NHNE cells (1 × 10^5^ cells/well) were seeded into 12 Transwell inserts. After 24 h, LXA_4_, IL-4, and siFPRL1 (each 1nM/L, 10μl/mL and 25nM/mL) were mixed with 1 µL lipofectamine, added to the cells, and incubated for 48 h.

### RNA preparation and gene expression analyses

The TRIzol reagent (Invitrogen) was used to isolate total RNAs from BEAS2B and NHNE cells after 48 h of LXA_4_/IL-4/siFPRL1 treatment. Real-time PCR was performed using the LightCycler FastStart DNA Master SYBR Green Kit (Roche Applied Science) and 0.5 μL amounts of cDNA (corresponding to 25 ng of total RNA) in a final volume of 10 μL 2.5 mM MgCl_2_ with 0.5 μM of each primer. PCR was performed using a LightCycler (45 cycles of 95 ºC for 10 s, annealing at a specific temperature for 5 s, and 72ºC for 10 s. The data were normalized to those of GAPDH. Melting curves were used to evaluate amplification specificity, according to the manufacturer's instructions (Roche Applied Science). The MUC5AC primers were F: 3ʹ-GCTCATCCTAAGCGACGTCT-5ʹ and R: 3ʹ-GGGGGCA TAACTTCTCTTGG-5 ʹ.

### Western blotting

Cells were collected and lysed for mucins and the followed immunoblotting as previously described. Western blotting was used to analyse the expression of P38, ERK, and JNK. BEAS2B cells was homogenized in lysis buffer (150 mM NaCl, 1.0% [v/v] nonyl phenoxypolyethoxylethanol-40 and 50 mM Tris HCl; Elpis Bio, Daejeon, Korea) containing a protease inhibitor (Sigma-Aldrich, St Louis, MO, USA) and clarified by centrifugation at 13,000 rpm for 15 min at 4ºC. Protein concentration of supernatant was quantitated by BCA protein assay kit (Pierce Biotechnology). The crude proteins (35 mg/lane) were separated in SDS-PAGE, and then transferred onto nitrocellulose membrane (Millipore, Bedford, MA). The supernatant proteins were resolved by 7.5% (w/v) sodium dodecyl sulphate-polyacrylamide gel electrophoresis (SDS-PAGE, Bio-Rad, Hercules, CA, USA) and transferred to a polyvinylidene difluoride membrane over 2 h at 300 mA. The membrane was blocked with 5% (w/v) bovine serum albumin (BSA) for 2 h at room temperature and incubated with primary antibodies against P38, P-ERK, and P-JNK (Cell Signaling Technology); and rabbit-beta-actin (Sigma-Aldrich) at 4°C overnight. The membranes were incubated with horseradish peroxidase-conjugated secondary antibodies (Abcam, Cambridge, UK) and proteins were detected using an LAS-4000 imaging system (Fujifilm, Tokyo, Japan). Band intensities were determined using Image J software (NIH, Bethesda, MD, USA).

### Immunofluorescence study

Tissue preparation was followed by fixation, dehydration, embedding, and staining. Tissues were fixed overnight at 25°C in 4% (v/v) paraformaldehyde (Biosesang, Seoul, Korea) and then underwent tissue processing (TP1020; Leica Biosystems, Wetzlar, Germany), dehydration through a series of graded ethanol baths, wax infiltration, and paraffin embedding (EG1150; Leica Biosystems). Sections (4-μm-thick) were prepared using a microtome (Leica Biosystems) and mounted on poly-L-lysine-coated microscopic slides (Mutokagaku, Tokyo, Japan). After rehydration, slides were blocked with 1% (w/v) BSA for 1 h and incubated overnight at 4°C with 300 μL of primary anti-FPR1/FPR2/IL-4Ra antibodies diluted in DAKO antibody diluent (including 1% [v/v] of the background reducing agent). The next day, secondary antibody was added and the suspensions was held at room temperature for 30 min. After washing with Tween-20 (TTBS), DAPI was added and incubation continued at room temperature for 2 min.

### Statistics

“Resource equation method” was used to determine sample size because it was impossible to assume the effect size or no previous published studies for power analysis. With the formula for the resource equation method (E = Total number of sample - Total number of groups, any sample size, which keeps E between 10 and 20, should be considered to be adequate.), total number of sample between 10 and 20 for an effect of LXA4 was adequate for sample size determination.

Data were analysed using IBM SPSS Statistics (ver. 22.0; IBM Corp., Armonk, NY, USA) and GraphPad Prism software (ver. 6.0; GraphPad Software Inc., La Jolla, CA, USA). Data are presented as mean ± standard deviation. The unpaired Student t-test or one-way analysis of variance was used to determine the significance of between-group differences. A P-value <0.05 was considered to indicate significance.

## Results

### Lipoxin A4 suppressed IL-4 induced MUC5AC and MUC5B gene expression in airway epithelial cells

**Figure 1:**
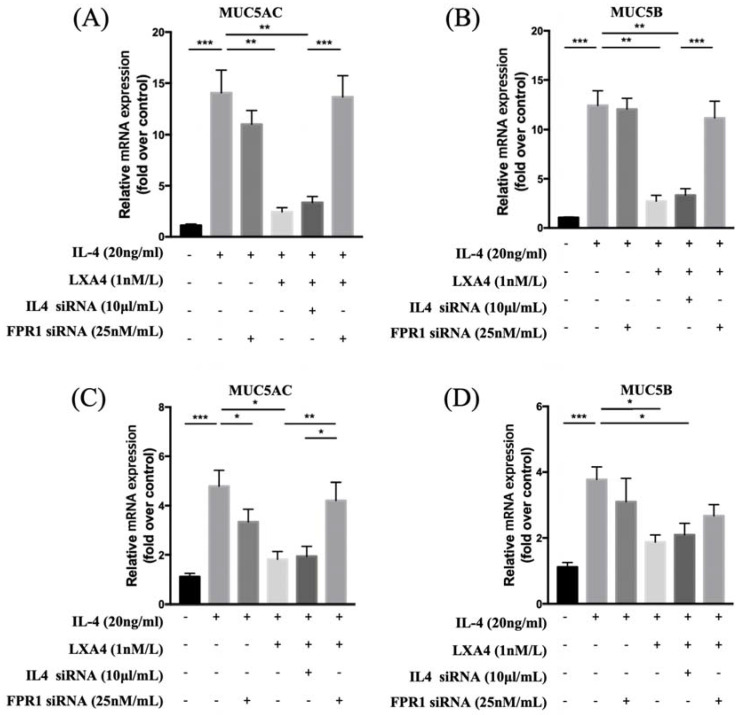
Suppression of IL-4-induced MUC5AC and 5B gene activation by LXA_4_ in airway epithelial cells (A, B) and NHNE cells (C, D). (n = 5 donors of NHBE; grown in duplicate) *p<0.05 compared to control.

To evaluate the expression of MUC 5AC and 5B, we treated cells with IL-4 at various concentrations (0, 5, 10, and 20 ng/mL) for 24 h and subjected cell lysates to RT-PCR. When NHNE cells were treated with various doses of IL-4 for different times, MUC5AC mRNA expression was up-regulated in a dose- and time-dependent manner, suggesting that IL-4 up-regulates MUC5AC and MUC5B mRNA expression. IL-4 affected MUC5AC and MUC5B expression in a dose-dependent manner without cytotoxicity. We used 20 ng/mL IL-4 in the following experiments. Exposure of BEAS-2B cells to IL-4 for 0, 12, 24, and 48 h showed that IL-4 stimulated MUC5AC and MUC5B expression in a time-dependent manner. The increase in MUC5AC mRNA expression was higher than that of MUC5B mRNA. MUC5AC expression increased significantly between 24 and 72 h after IL-4 stimulation. MUC5B expression increased significantly at 24 h after IL-4 stimulation (Figure S1). In addition, we performed western blot analysis to evaluate whether IL-4 up-regulates MUC5B protein expression in NHNE cells. Interestingly, MUC5B expression peaked at 48 h. (data not shown). IL-4-induced MUC5AC and MUC5B expression decreased when LXA4 was added. Inhibition of lipoxin receptor expression by siFPRL1 pre-treatment increased MUC5AC and MUC5B expression compared to the control in BEAS-2B cells (Figure 1A, B). We next investigated how LXA_4_ affected mucin expression in cultured NHNE cells; the response was modest. IL-4 upregulated MUC5AC and MUC5B mRNA expression, but this was suppressed by LXA_4_. Inhibition of the lipoxin receptor by siFPRL1 pre-treatment increased MUC5AC and MUC5B mRNA levels (Figure 1C, D).

### Expression of MUC5AC and MUC5B in airway epithelial cells

**Figure 2:**
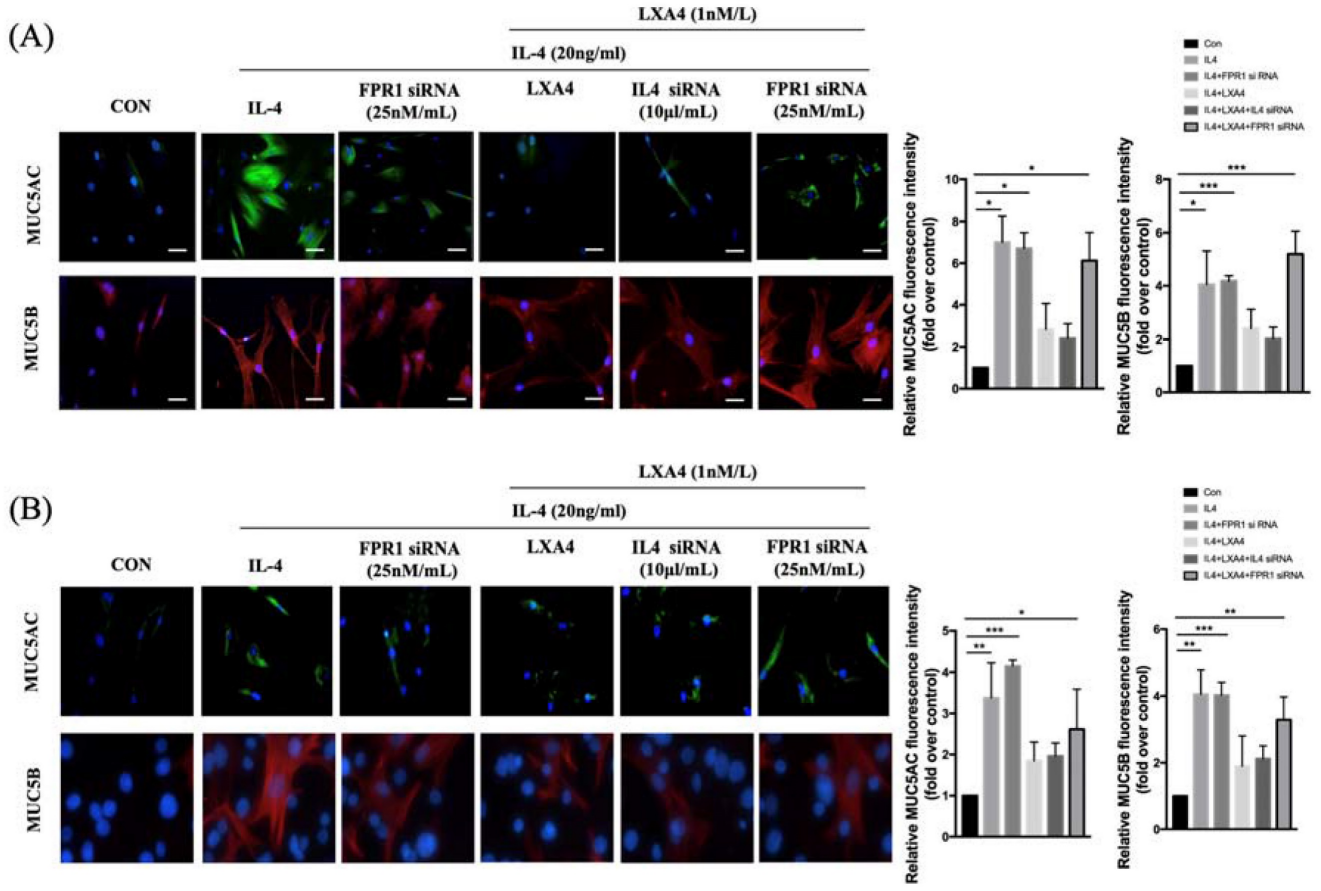
Expression of MUC5AC and 5B as determined by immunofluorescence staining in airway epithelial cells (A) and NHNE cells (B). All experiments were performed using 5 donors, grown in duplicate, with 3-6 wells per condition. *p<0.05 compared to control. DAPI staining of the nuclei showed similar number of cells in all conditions (data not shown). Green: MUC5AC; red: MUC5B. (scale bar = 50 μm).

To further explore the intracellular effects of LXA_4_ on airway epithelial cells, we stained MUC5AC and MUC5B in BEAS-2B and NHNE cells treated with IL-4 (Figure 2A,C). BEAS-2B and NHNE cells were stimulated with IL-4 for 48 h; fluorescence intensity increased compared to control cells (by 7.2-and 4.9-fold in MUC5AC and MUC5B, respectively, in BEAS-2B airway epithelial cells, P=0.05, Figure 2B; and by 3.4- and 4.0-fold, respectively, in NHNE cells, P<0.05, Figure 2D). LXA_4_ significantly reduced MUC5AC and MUC5B expression by BEAS-2B cells (by 2.8- and 2.6-fold compared to control, P<0.05, Figure 2B). In NHNE cells, IL-4-induced MUC5AC and MUC5B expression was also inhibited by LXA_4_ (by 1.8- and 1.9-fold, respectively, compared to control; P<0.05; Figure 2D). Thus, IL-4-induced MUC5AC and MUC5B protein expression was inhibited by LXA_4_. MUC5AC and MUC5B expression was restored by siFPRL1 in BEAS-2B and NHNE cells.

### LXA_4_ suppressed IL-4-induced MUC5AC and MUC5B protein expression

**Figure 3:**
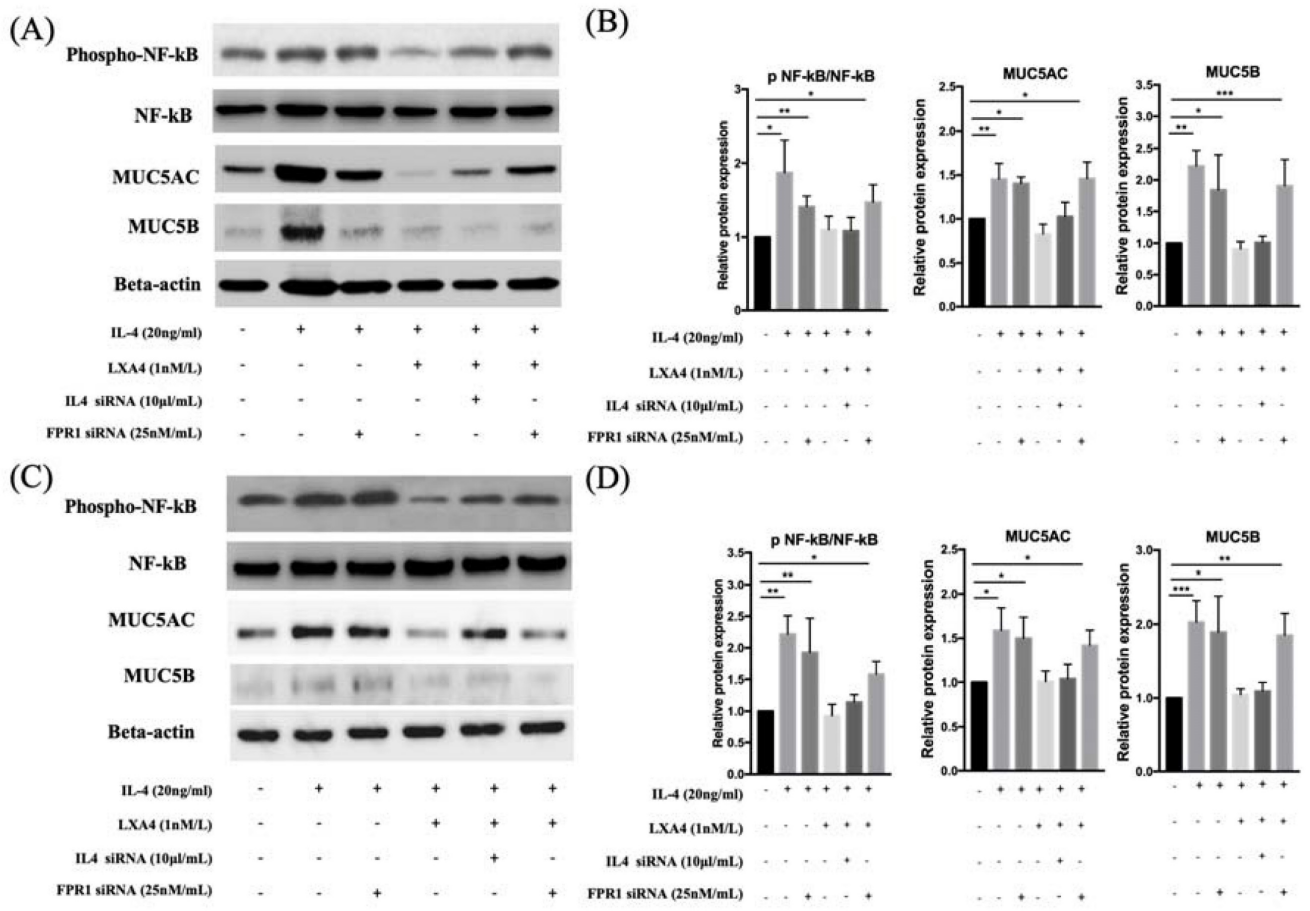
LXA_4_ inhibits IL-4-induced activation of MUC5AC and MUC5B protein synthesis in airway epithelial cells (A, B) and NHNE cells (C, D). All experiments were performed using 5 donors, grown in duplicate, with 3-6 wells per condition. *p<0.05 compared to control.

MUC5AC and MUC5B expression increased in IL-4 (20 ng/mL)-treated groups compared to controls; the expression was decreased by LXA_4_. Inhibition of lipoxin receptor expression by siFPRL1 increased MUC5AC and MUC5B expression compared to controls (Figure 3A, B). IL-4-induced MUC5AC and MUC5B expression was restored by siFPRL1. To explore the downstream signals of IL-4 in BEAS-2B and NHNE cells, we investigated activity in the NF-κB pathway. In BEAS-2B cells, IL-4 significantly increased NF-kB phosphorylation; this was inhibited by LXA_4_ in Figure 3 (A). Thus, NF-kB was involved in the regulation of MUC5AC and MUC5B expression. In NHNE cells, IL-4-induced MUC5AC and MUC5B expression was also inhibited by LXA_4_ (Figure 3C, D). Also, IL-4 increased NF-kB phosphorylation; this was inhibited by LXA_4_.

### LXA_4_ inhibited the activation of ERK 1/2 and p38 in the MAP kinase pathway

**Figure 4:**
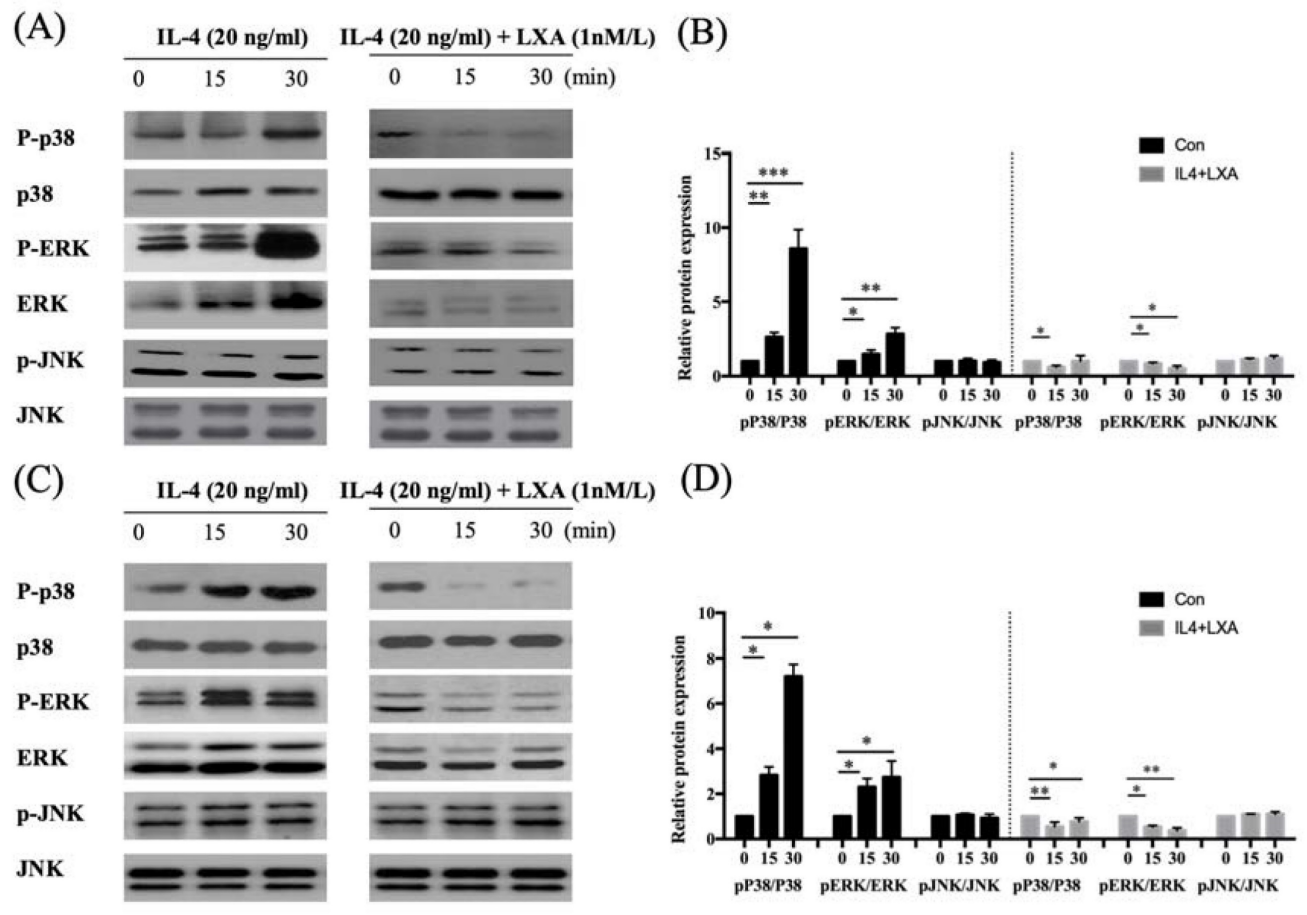
LXA_4_ suppresses p38 and ERK signalling in airway epithelial cells (A, B) and NHNE cells (C, D). All experiments were performed using 5 donors, grown in duplicate, with 3-6 wells per condition. *p<0.05 compared to control.

**Figure 5:**
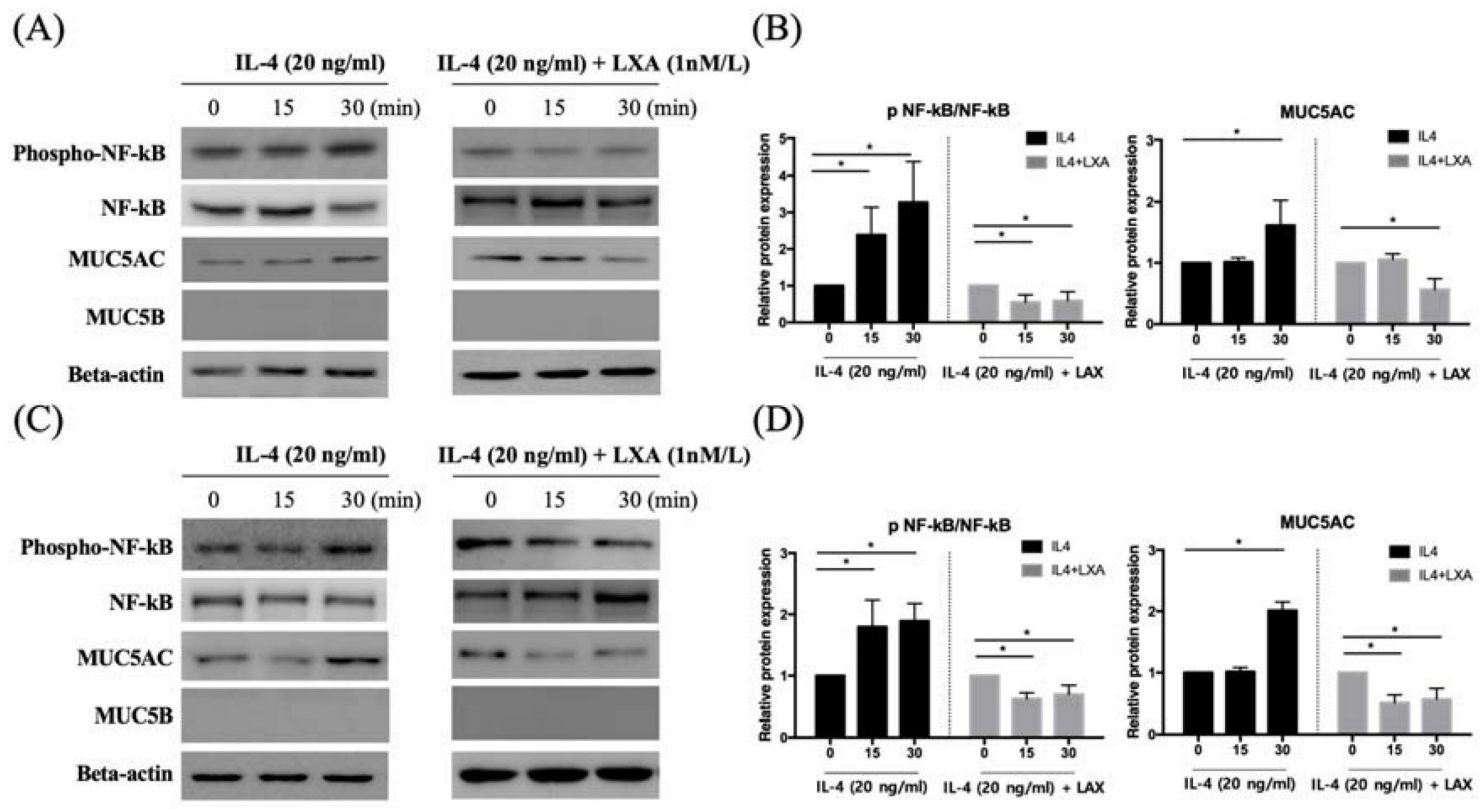
LXA_4_ inhibits IL-4-induced activation of MUC5AC and MUC5B protein synthesis in airway epithelial cells (A, B) and NHNE cells (C, D). All experiments were performed using 5 donors, grown in duplicate, with 3-6 wells per condition. *p<0.05 compared to control.

NF-kB activation triggers mitogen-activated protein kinase (MAPK) signalling. To establish whether the decrease in MUC5AC expression induced by IL-4 affected NF-kB MAPK (p38, ERK, and JNK) signalling, we pre-treated cells with LXA_4_ and used Western blotting to measure the levels of p-ERK, p38, and p-JNK. LXA_4_ inhibited the phosphorylation of both p38 and ERK, suggesting that MAPK lay downstream of the NF-κB signal transduction pathway in cells in which IL-4 induced MUC5AC and MUC5B expression. However, JNK was not affected by LXA_4_ in BEAS-2B cells (Figure 4A, B). In NHNE cells, LXA_4_ suppressed the phosphorylation of both p38 and ERK, but not of JNK (Figure 4C, D). Exposure of BEAS-2B and NHNE cells to IL-4 for 0, 15, and 30 min showed that IL-4 stimulated MUC5AC expression in a time-dependent manner. MUC5AC and NF-kB expression was suppressed by LXA_4_. However, cultures subjected to short-term IL-4 stimulation did not produce MUC5B (Figure 5A, B).

### LXA_4_ and IL-4 receptor levels in nasal mucosa

**Figure 6:**
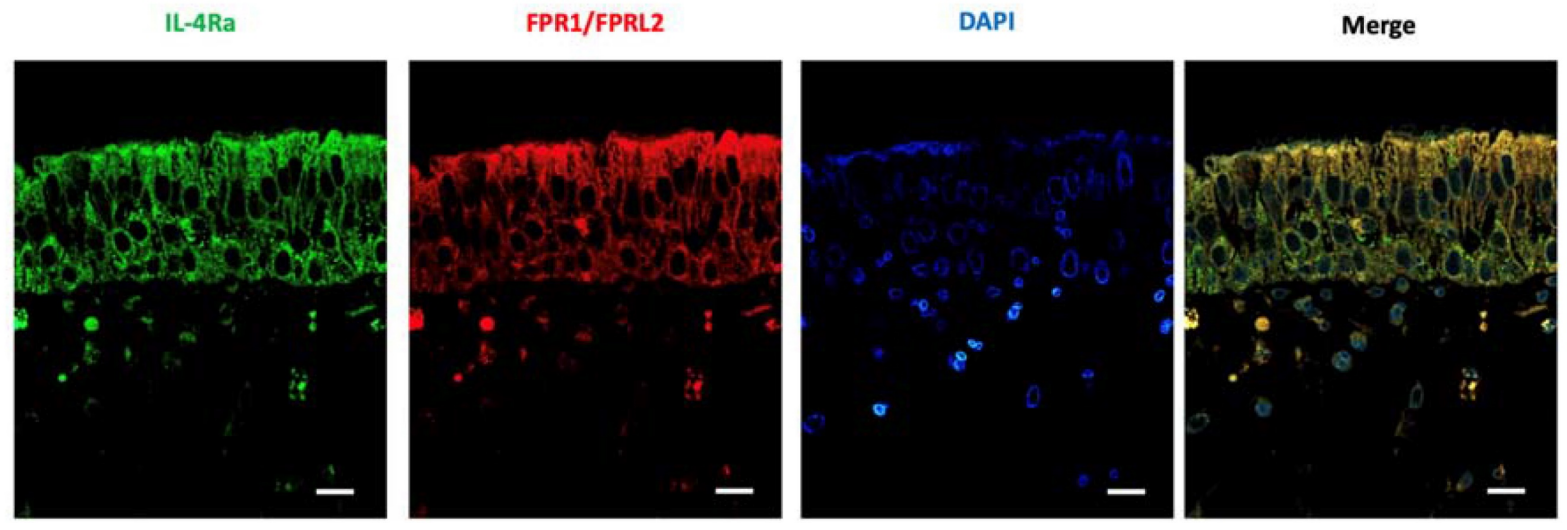
Expression of IL4 and LXA_4_ receptors (as revealed by immunofluorescence) in the nasal mucosa of representative patients with chronic rhinitis. The fluorescence intensities of IL4 and LXA_4_ receptors were used as representative images (scale bar = 50 μm).

Nasal mucosa was subjected to immunofluorescence staining to determine the expression patterns of LXA_4_ and the IL-4 receptor, which were shown to be co-expressed in nasal epithelial cells (Figure 6).

## Discussion

We found that LXA_4_ suppressed IL-4-induced MUC5AC and MUC5B expression in a dose-dependent manner. These decreases were mediated by the IL-4 receptor and MAPK pathway, including both phospho-ERK and phospho-p38 MAPK. NFκB phosphorylation was inhibited in BEAS-2B and normal nasal epithelial cells.

Airway epithelial cell inflammation is associated with mucin overexpression (particularly of MUC5AC and MUC5B) and hypersecretion 15,16. Mucus is important both physiologically and pathologically 17. A detailed understanding of how airway mucin expression is regulated by external stimuli or cytokines may yield new therapeutic strategies for inhibition of mucus hypersecretion. We found that, in human BEAS-2B cells and primary cultures of normal nasal epithelial cells, IL-4 increased MUC5AC and MUC5B expression. Dabbagh et al. reported that IL-4 induced mucin gene expression and mucous glycoconjugate production both in vitro and in vivo 18. However, although MUC5AC expression was significantly increased by IL-4 in vivo, this was not the case in vitro.

LXs are pro-resolving mediators. in animal models, bioactive stable LXA_4_ analogues have been shown to inhibit airway hyper-responsiveness and allergic inflammation, including eosinophil trafficking and accumulation in tissues 5. LXs are produced in the upper respiratory tract, including nasal polyps 19, and are found in the nasal lavage fluid of aspirin-challenged patients with aspirin-exacerbated respiratory disease (AERD) 20. LXs are also present in the lower airway bronchoalveolar lavage fluid (BALF) of patients with asthma 1. Perez-Novo et al. reported that LXA_4_ levels were significantly higher in patients with chronic rhinitis than controls, and lower in aspirin-sensitive than aspirin-tolerant subjects 3. However, little is known about the effects of LXA_4_ on mucins of the nasal mucosa. We explored whether LXA_4_ inhibited excess airway mucus production. As expected, IL-4 markedly upregulated MUC5AC and MUC5B expression. However, these increases were suppressed by LXA_4_ in BEAS-2B and NHNE cells. Thus, LXA_4_ may affect IL 4-induced mucin expression. Blockade of LXA_4_ induction by siFPRL1 suppressed mucus overproduction by airway epithelium. In addition, in both BEAS-2B and NHNE cells, IL-4-induced MUC5AC and MUC5B protein expression was inhibited by LXA_4_, as revealed by Western blotting. Within 48 h after the addition of IL-4, increased MUC5AC and MUC5B mRNA and protein levels were evident. A recent study found showed that transgenic mice overexpressing IL-4 accumulated mucus glycoproteins in the airways and overexpressed MUC5AC mRNA in the lungs 21.

We found that IL-4-induced MUC5AC and MUC5B expression was mediated by the MAPK pathway (both phospho-p38 MAPK and phospho-ERK). LXA_4_ decreased IL-4-induced MUC5AC and MUC5B expression via MAPK signalling. In general, this signalling plays an important role in regulating cellular responses to external stimuli. Wong et al. found that IL-4 and IL-13 activated p38 MAPK, ERK, and JAK-2, but not JNK, in BEAS-2B cells 22. Diandian et al. showed that, on exposure of BEAS-2B cells to cigarette smoke, airway inflammation increased via the ERK and p38 kinase pathways 23.

Previous studies found that the MAPK signalling pathway was involved in IL-1β-induced MUC5AC mRNA overexpression 25-27. MAPKs tightly control cellular responses to various stimuli (mitogens, osmotic stress, heat shock protein, and proinflammatory cytokines). The effects of these stressors on IL-4-mediated proinflammatory signalling require investigation.

IL-4 induced p38 and ERK phosphorylation within 30 min in BEAS-2B cells; LX4 significantly suppressed p38/ERK MAPK pathway activity. We confirmed that LX4 inhibited p38 and ERK phosphorylation, in turn reducing mucin expression by BEAS-2B and normal nasal epithelial cells. Various LXs reduced ERK and p38 MAPK phosphorylation 28.

We found that IL-4 activated the NF-kB pathway. Similarly, Jayawickreme et al. reported that IL-4 activated NF-κB 29,30. Fujisawa et al. showed that NF-κB-based transcription was involved in IL-1β- and IL-17A-mediated MUC5AC regulation in airway epithelium 33.

MAPK signalling activates NF-kB signalling. We found that LXA_4_ pre-treatment attenuated IL-4 induced airway inflammation, possibly via changes in MAPK-NF-κB signalling. Wong et al. showed that exposure of BEAS-2B cells to allergic inflammation was associated with activation of the p38 MAPK and NF-kB pathways 22. LXA_4_ reduced the nuclear translocation of NF-kB in human neutrophils, mononuclear leukocytes, and macrophages 31,32. Wang et al. found that LXA_4_ attenuated the lipopolysaccharide (LPS)-induced pro-inflammatory response by inhibiting activation of MAPK and NF-kB in BV-2 microglial cells, but did not affect JNK phosphorylation. Kure et al. showed that LXA_4_ reduced LPS-induced inflammation in macrophages and intestinal epithelial cells by inhibiting NF-kB activation 32.

Notably, we found that that short-term (30-min) exposure of BEAS-2B cells to IL-4 increased the expression levels of MUC5AC and NF-kB. Similarly, Jiang et al. showed that IL-4 activated NF-κB 29. We found that short-term (30-min) exposure to IL-4 did not stimulate MUC5B expression in normal nasal epithelial cells, but MUC5B expression was moderately induced by long-term (24-h) IL-1β expression, as previously reported by Fujisawa et al. 33.

To explore the effects of LXA_4_ on nasal mucosa, we first determined that the tissue expressed the LXA_4_ receptor. The IL-4R and LXA_4_ receptors were co-expressed in epithelial and submucosal nasal tissues. The LXA_4_ receptor is expressed in the lung 34, kidney 35, stomach 36, goblet cells 37, and cornea 38, and mediates anti-inflammatory activity. In conclusion, IL-4 induces MUC5AC and MUC5B gene expression by activating the ERK and p38 MAP kinases. LXA_4_ is a potent inhibitor of IL-4-induced MUC5AC and MUC5B gene expression. The mechanism involves inhibition of ERK and p38 MAP kinase phosphorylation, and downstream transcriptional factors, in human airway epithelial cells.

## Supplementary Material

Supplementary figure.
