# Supplementary material for: Exogenous Lipoxin A4 attenuates IL4-induced Mucin Expression in Human Airway Epithelial Cells
Source: Int J Med Sci. 2023 Feb 5;20(3):406–14. doi: 10.7150/ijms.79525 (PMC9969498; doi:10.7150/ijms.79525)
Supplement: Supplementary file 1 — Supplementary figure. [file ijmsv20p0406s1.pdf]

Supplemental Figure S1.

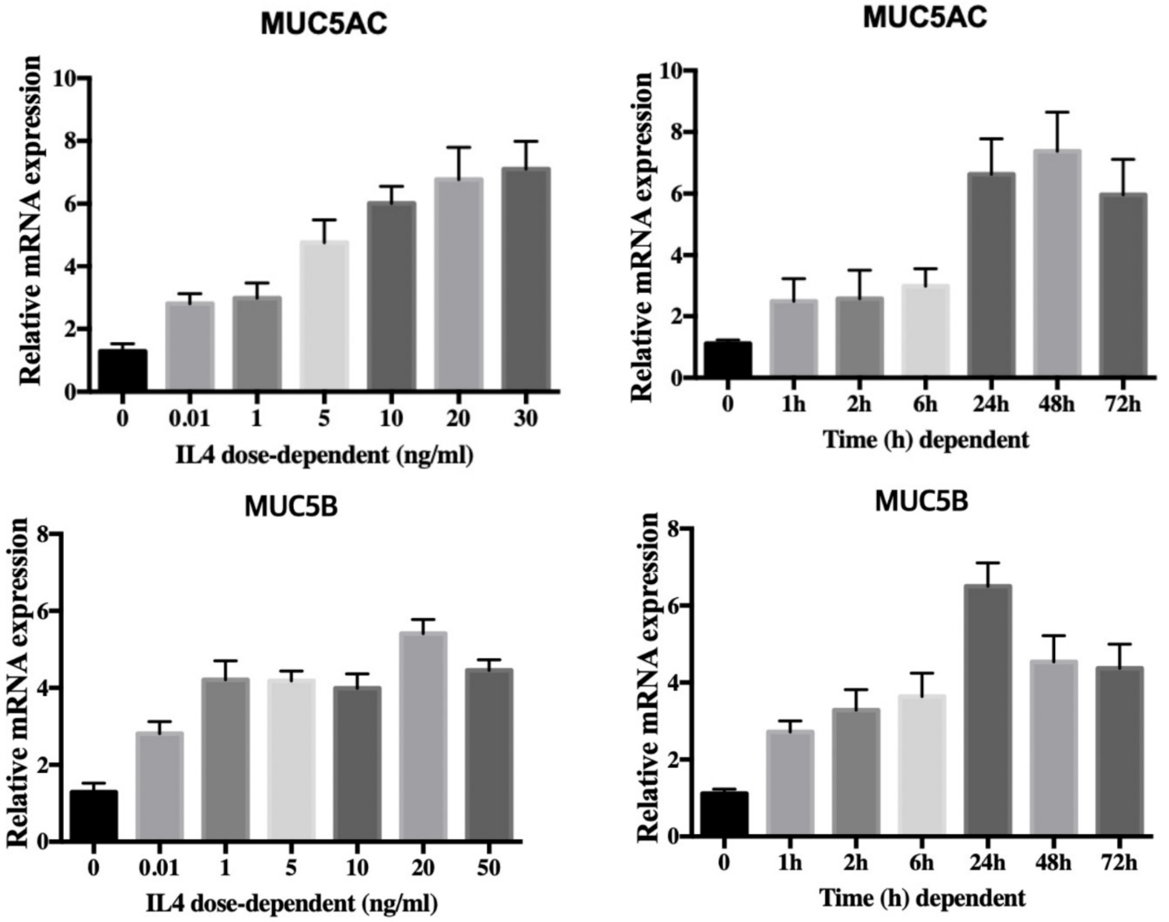

Figure S1. Dose and time-dependent upregulation of MUC5AC and MUC5B mRNA expression by interleukin-4 (IL-4) in NHNE cells.
